# Left Atrial 4D Blood Flow Dynamics and Hemostasis following Electrical Cardioversion of Atrial Fibrillation

**DOI:** 10.3389/fphys.2017.01052

**Published:** 2017-12-12

**Authors:** Merih Cibis, Tomas L. Lindahl, Tino Ebbers, Lars O. Karlsson, Carl-Johan Carlhäll

**Affiliations:** ^1^Division of Cardiovascular Medicine, Department of Medical and Health Sciences, Linköping University, Linköping, Sweden; ^2^Center for Medical Image Science and Visualization, Linköping University, Linköping, Sweden; ^3^Division of Microbiology and Molecular Medicine, Department of Clinical and Experimental Medicine (IKE), Linköping University, Linköping, Sweden; ^4^Departments of Cardiology, Medical and Health Sciences, Linköping University, Linköping, Sweden; ^5^Departments of Clinical Physiology, Medical and Health Sciences, Linköping University, Linköping, Sweden

**Keywords:** atrial fibrillation, cardioversion, atrial stunning, 4D flow CMR, hemostasis, stasis

## Abstract

**Background:** Electrical cardioversion in patients with atrial fibrillation is followed by a transiently impaired atrial mechanical function, termed atrial stunning. During atrial stunning, a retained risk of left atrial thrombus formation exists, which may be attributed to abnormal left atrial blood flow patterns. 4D Flow cardiovascular magnetic resonance (CMR) enables blood flow assessment from the entire three-dimensional atrial volume throughout the cardiac cycle. We sought to investigate left atrial 4D blood flow patterns and hemostasis during left atrial stunning and after left atrial mechanical function was restored.

**Methods:** 4D Flow and morphological CMR data as well as blood samples were collected in fourteen patients at two time-points: 2–3 h (Time-1) and 4 weeks (Time-2) following cardioversion. The volume of blood stasis and duration of blood stasis were calculated. In addition, hemostasis markers were analyzed.

**Results:** From Time-1 to Time-2: Heart rate decreased (61 ± 7 vs. 56 ± 8 bpm, *p* = 0.01); Maximum change in left atrial volume increased (8 ± 4 vs. 22 ± 15%, *p* = 0.009); The duration of stasis (68 ± 11 vs. 57 ± 8%, *p* = 0.002) and the volume of stasis (14 ± 9 vs. 9 ± 7%, *p* = 0.04) decreased; Thrombin-antithrombin complex (TAT) decreased (5.2 ± 3.3 vs. 3.3 ± 2.2 μg/L, *p* = 0.008). A significant correlation was found between TAT and the volume of stasis (*r*^2^ = 0.69, *p* < 0.001) at Time-1 and between TAT and the duration of stasis (*r*^2^ = 0.34, *p* = 0.04) at Time-2.

**Conclusion:** In this longitudinal study, left atrial multidimensional blood flow was altered and blood stasis was elevated during left atrial stunning compared to the restored left atrial mechanical function. The coagulability of blood was also elevated during atrial stunning. The association between blood stasis and hypercoagulability proposes that assessment of left atrial 4D flow can add to the pathophysiological understanding of thrombus formation during atrial fibrillation related atrial stunning.

## Introduction

Atrial fibrillation (AF) is the most common sustained arrhythmia, with an increasing prevalence with age, presence of cardiovascular risk factors and cardiovascular diseases (Odutayo et al., 2016; Xu et al., 2016). The lack of normal mechanical contraction in the left atrium (LA) and left atrial appendage (LAA) leads to stasis of blood, elevating the thromboembolic risk and consequently the risk of stroke in AF patients (Iwasaki et al., 2011). Depending on the type of AF and the severity of associated symptoms, several treatment options are considered, including electrical cardioversion (Xu et al., 2016). Electrical cardioversion is a standard rhythm-focused treatment which restores electrical and mechanical function of the atria by delivering an electrical shock to the heart. Following cardioversion, LA mechanical function gradually recovers. The period of impaired LA mechanical function, is a condition termed atrial stunning (Khan, 2003). Left atrial stunning lasts a few weeks, being at its maximum immediately after cardioversion with progressive improvement of atrial function thereafter. Although the exact mechanism leading to atrial stunning is not entirely known, studies suggest that it is caused by changes in atrial myocardium during AF and is influenced by the duration of AF, atrial size and underlying structural heart disease (Watson et al., 2009).

The risk of post-cardioversion thromboembolism persists during atrial stunning and decreases gradually by restoration of atrial mechanical activity (Khan, 2003). It is likely that the increased risk of atrial thrombus formation during atrial stunning is associated with abnormal blood flow patterns caused by the impaired atrial mechanical function, despite reversion to sinus rhythm. However, widely used blood flow measurement techniques such as Doppler echocardiography are not sufficient for detailed evaluation of the three-dimensional (3D) blood flow patterns in the atria, as this ultrasound technique permits only one single velocity component. Alternatively, 4D Flow CMR permits comprehensive measurements of three velocity components in a 3D volume (Dyverfeldt et al., 2015), and hence, can provide multidimensional flow measures from the entire 3D atrial volume (Arvidsson et al., 2013; Föll et al., 2013). Recent studies based on 4D Flow CMR have shown promising results and added to flow-specific pathophysiological understanding of AF (Fluckiger et al., 2013).

In this longitudinal study, we investigated patients 2–3 h and 4 weeks following electrical cardioversion of AF. Specifically, we sought to investigate LA 4D flow patterns and hemostasis at LA stunning as well as at restored LA mechanical function. Further, we hypothesized that there is a correlation between abnormal LA 4D flow patterns and increased coagulability during atrial stunning.

## Methods

### Study population

Twenty-seven patients with persistent AF were included in the study and underwent electrical cardioversion. CMR data and blood samples were collected at two different time points following cardioversion: 2–3 h (from here on referred to Time-1) and 4 weeks (from here on referred to Time-2). All patients gave informed consent prior to participation and the study was approved by the Regional Ethical Review Board (Dnr 2014/414-31).

Inclusion criteria were: patients with persistent AF scheduled for electrical cardioversion with an AF duration of 3–20 weeks (up until cardioversion). The patients were treated with periprocedural anticoagulants according to guidelines: apixaban (Eliquis®), dabigatran (Pradaxa®), rivaroxaban (Xarelto®), or warfarin (Warfarin Orion®) (Kirchhof et al., 2016).

Exclusion criteria were: (1) uncontrolled hypertension (>160/95 mmHg), (2) Left ventricular (LV) ejection fraction <40%, (3) age > 80 years, (4) previous ablation or heart surgery, (5) more than mild to moderate valvular disorder in the aortic- or mitral valve, (6) more than moderate dilatation of the left ventricle, (7) contraindications for MRI-examination, (8) failure of converting AF to sinus rhythm, (9) relapse in AF that were not converted within 48 h, occurring in the course of 4 weeks following the cardioversion, and (10) LV inflow vs. outflow difference exceeding 15%, as an indicator of data quality.

### Acquisition of CMR data

The CMR data was acquired using a clinical 3T scanner (Philips Ingenia, Philips Medical Systems, Best, the Netherlands) and the protocol included time-resolved, 3D phase-contrast (4D Flow) and bSSFP (morphological) data. The 4D flow data were acquired during free breathing using retrospective ECG and respiratory gating and the settings included: velocity encoding (VENC) 120 cm/sec, flip angle 5°, repetition time 5.1 ms, echo time 3 ms, echo train length 2, spatial resolution 2.8 × 2.8 × 2.8 mm^3^, field of view 300 × 300 × 120 mm^3^, and parallel imaging (SENSE) with a reduction factor of 3. The data was reconstructed into 40 time frames and was corrected for eddy-current distortions using a weighted fourth order fit to static tissue and phase wrapping using a temporal phase unwrapping method (Xiang, 1995). The bSSFP data were acquired in standard long-axis two-, three-, and four-chamber views as well as short-axis images. The morphological images were acquired with retrospective cardiac gating using the following setting: flip angle 45°, repetition time 2.8 ms, echo time 1.4 ms. All morphological images were acquired in 30 time frames with a slice thickness of 8 mm. The long-axis and short-axis images were reconstructed with a resolution of 0.9 × 0.9 mm^2^ and 0.83 × 0.83 mm^2^ respectively.

### Collection and analyses of blood samples

Whole blood samples were collected in vacuum tubes containing 1/10 volume of 0.109 M citrate and centrifuged at 2,500 × g for 20 min. Plasma was then withdrawn and stored in −70°C until further analysis. Thrombin-antithrombin complex (TAT) as a marker of coagulation activation was measured with ELISA with reagents from Siemens (Marburg, Germany), human sP-selectin as a marker of platelet activation with ELISA from R&D Systems (Abingdon, UK), D-Dimer as a marker of coagulation and fibrin formation with latex-enhanced turbidimetry with reagents from Medirox (Nyköping, Sweden) on a Sysmex instrument CS2000i purchased from Siemens (Stockholm, Sweden) and the marker of endothelial function von Willebrand Factor activity (vWF) with reagents from Siemens on a Sysmex instrument CS2000i.

### Post-processing of 4D flow CMR data

The 4D Flow CMR magnitude and velocity data were combined to obtain 4D Phase Contrast Magnetic Resonance CardioAngiography (PC-MRCA) (Bustamante et al., 2016). The PC-MRCAs are the improved, time-resolved phase contrast magnetic resonance angiograms obtained by registering different time frames. The time frames of early diastole (ED) and late diastole (LD) were defined as two consecutive peak velocities observed in the vicinity of the mitral valve and determined using 4D Flow CMR data. If peak flow was absent at LD in Time-1 due to atrial stunning, we defined the late diastolic time frame by assuming that the number of time frames between ED and LD was equal during CMR scans in Time-1 and Time-2. The time-resolved LA volume was manually segmented on PC-MRCAs using an open source software ITK-SNAP. The LA volumes were calculated using the manual volumetric segmentations performed on the PC-MRCAs. In order to evaluate LA stunning, the LA mechanical function was measured by calculating maximum change in LA volume (ΔVolume_max_) which is defined as the difference between maximum and minimum LA volume divided by maximum LA volume within one cardiac cycle.

**Figure 1:**
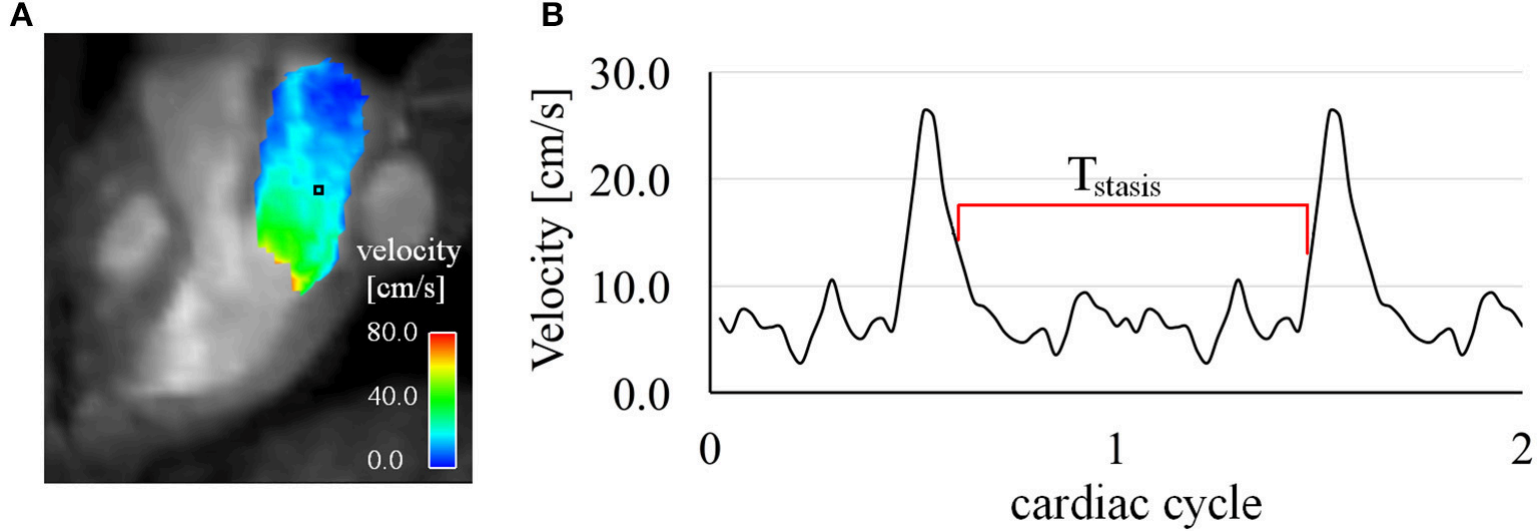
**(A)** PC-MRCA of a representative subject and the velocity distribution in a cross-section within the LA (corresponding to a 3-chamber long-axis view) during early diastole at Time-1. **(B)** Velocity waveform of a voxel (shown in black box in panel **A**) inside LA. The duration of stasis (T_stasis_) is calculated as the longest continuous period with velocities below 15 cm/s normalized by the number of cardiac time frames.

Mean velocity (V_mean_), peak velocity (V_peak_) (99th percentile), of the LA were calculated at ED and LD for Time-1 and Time-2. We also calculated mean vorticity (ω_mean_), and near wall vorticity (ω_wall_), which are measures of the local rotation of flow providing information about local flow patterns. We quantified ω_wall_ by calculating the mean vorticity of the voxels adjacent to the walls of LA volume. In order to quantify blood stasis in a more advanced way, all time frames of PC-MRCA and 4D Flow CMR were non-rigidly registered to the early-diastolic time frame resulting in images of motionless heart. Using the registered 4D flow data, we calculated the duration of blood stasis for every LA voxel by determining the longest continuous period with blood velocities below 15 cm/s normalized by the total number of cardiac time frames (T_stasis_). Figure 1A shows the PC-MRCA of a representative patient and the velocity magnitude distribution in one cross-section within the LA at ED. Figure 1B shows the T_stasis_ of one of the voxels inside LA. We calculated volume of stasis by determining the volume fraction of the LA with velocities below 15 cm/s throughout the entire cardiac cycle (Volume_stasis_) (Markl et al., 2016c). The non-rigid registration was performed on a desktop station using an in-house software package implemented in MATLAB (The MathWorks, Inc., Natick, Massachusetts, United States). The non-rigid registration was based on Morphon method and the registration parameters included linear interpolation, three scales and five iterations per scale (Knutsson and Andersson, 2005).

### Statistical analysis

The results are presented as mean ± 1 standard deviation. The changes in hemodynamic parameters and the markers of hemostasis between Time-1 and Time-2 were evaluated by paired student *t*-test. The Mann-Whitney *U*-test was used when the data was not normally distributed. Power analysis was performed for the intra-patient comparisons over time. The relation between stasis parameters and hemostasis markers were evaluated by linear regression analysis. The significance level of *p* < 0.05 was considered statistically significant. Statistical analyses were performed using the statistical software IBM SPSS v. 24 (IBM Corp, Armonk, NY).

## Results

### Study population

Five patients were excluded from the study due to persistent AF immediately after the electrical cardioversion procedure. Four patients were excluded due to relapse of AF during the 4 weeks of follow-up period after cardioversion. Furthermore, four patients were excluded due to LV inflow vs. outflow difference exceeding 15%.

The majority of the intra-patient comparisons over time were based on statistical analysis with acceptable power (at least ~80%). The patients (*n* = 14) had significantly higher heart rate (61 ± 7 vs. 56 ± 8 bpm, *p* = 0.01) and lower LV ejection fraction (53 ± 7 vs. 60 ± 5%, *p* < 0.001) at Time-1 compared to Time-2. Although the maximum LA volume did not change (121 ± 16 mL vs. 118 ± 22 mL, *p* = 0.30), the maximum to minimum LA volume (1.1 ± 0.1 vs. 1.4 ± 0.4, *p* = 0.002) and ΔVolume_max_ (8 ± 4 vs. 22 ± 15%, *p* = 0.009) increased significantly over time. The demographic details of the study population are presented in Table 1. Of note, all patients remained on the same medications at the Time-2 examination.

**Table 1:** Demographical and basic clinical data in all patients (*n* = 14).

	**Time-1**	**Time-2**	***p*-value**
Age (years)	65 ± 9		
Gender F/M	1/13		
Weight (kg)	95 ± 14		
Heart rate (bpm)	61 ± 7	56 ± 8	0.01
Blood pressure (mmHg)			
Systolic	137 ± 17	149 ± 20	0.06
Diastolic	88 ± 13	86 ± 12	0.38
LV end diastolic volume (mL)	186 ± 34	193 ± 38	0.28
LV end systolic volume (mL)	88 ± 23	76 ± 17	0.005
LV ejection fraction (%)	53 ± 7	60 ± 5	<0.001
LA max/min size	1.1± 0.1	1.4 ± 0.4	0.002
LA ΔVolume_max_ (%)	8 ± 4%	22 ± 15	0.009
Medication			
Beta blockers	14		
ACE-inhibitors or ARB	9		
Calcium antagonist	7		
Diuretics	4		
Lipid-lowering drugs	5		
Anticoagulant Warfarin/NOAC	5/9		

*LV, left ventricle; LA, left atrium; ΔVolume_max_, maximum change in LA volume; ACE, Angiotensin-converting enzyme; ARB, Angiotensin II receptor blockers; NOAC, New oral anticoagulants*.

**Figure 2:**
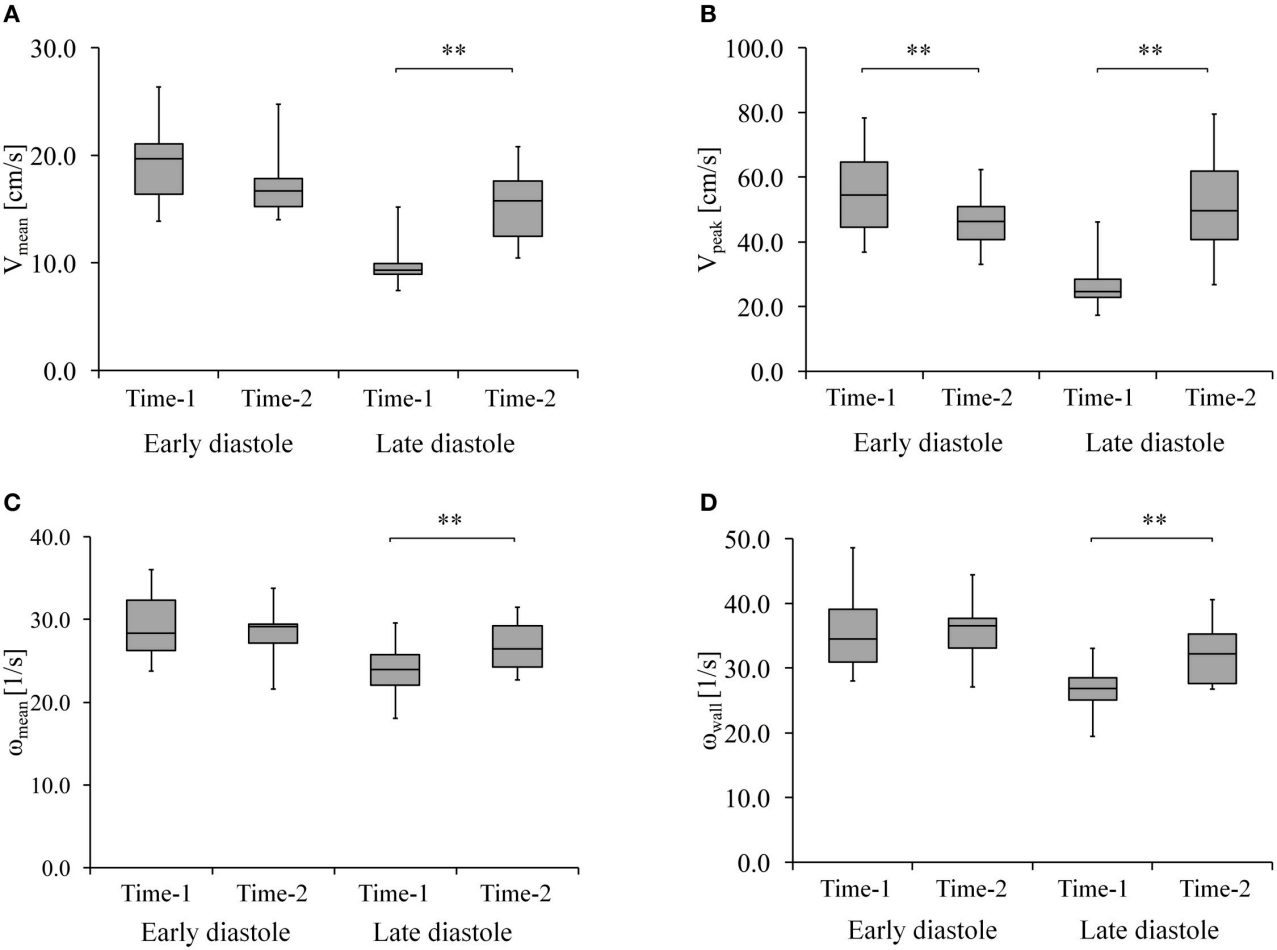
**(A)** Mean velocity (V_mean_) and **(B)** peak velocity (V_peak_) **(C)** mean vorticity of LA (ω_mean_) and **(D)** near wall vorticity (ω_wall_) at early and late diastole during Time-1 and Time-2. ^**^ Shows that *p* < 0.005.

**Figure 3:**
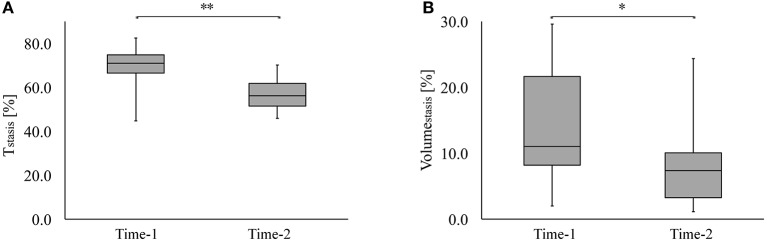
**(A)** The duration of stasis as a percentage of the cardiac cycle (T_stasis_) and **(B)** the volume of stasis (Volume_stasis_) as a percentage of LA volume during Time-1 and Time-2. ^**^ Shows that *p* < 0.005 and ^*^ shows that *p* < 0.05.

V_mean_ and V_peak_ at ED and LD are shown in Figures 2A,B. While V_mean_ at ED did not change over time (19 ± 4 vs. 17 ± 3 cm/s, *p* = 0.07), it showed a significant increase (10 ± 2 vs. 15 ± 3 cm/s, *p* < 0.001) at LD. V_peak_ decreased at ED (56 ± 13 vs. 46 ± 9 cm/s, *p* < 0.001) and increased at LD (27 ± 9 vs. 52 ± 15 cm/s, *p* < 0.001). Figures 2C,D show ω_mean_ and ω_wall_ at ED and LD. There was no significant difference in ω_mean_ (29 ± 4 1/s vs. 29 ± 3 1/s, *p* = 0.63) and in ω_wall_ (35 ± 6 1/s vs. 36 ± 4 1/s, *p* = 0.73) between Time-1 and Time-2 at ED. At LD, ω_mean_ (24 ± 3 1/s vs. 27 ± 3 1/s, *p* < 0.004) and ω_wall_ (27 ± 4 1/s vs. 32 ± 4 1/s, *p* < 0.001) increased. Figure 3 shows T_stasis_ and Volume_stasis_ during Time-1 and Time-2. Both T_stasis_ (68 ± 11 vs. 57 ± 8%, *p* = 0.002) and Volume_stasis_ (14 ± 9 vs. 9 ± 7%, *p* = 0.04) decreased over time.

The markers of hemostasis are reported in Table 2. TAT was found to be higher at Time-1 than at Time-2 (*p* = 0.001). Other hemostasis markers sP-selectin, D-dimer, and vWF did not change over time.

**Table 2:** The markers of hemostasis at Time-1 and Time-2.

	**Time-1**	**Time-2**	***p*-value**
TAT (μg/L)	5.2 ± 3.3	3.3 ± 2.2	0.008
Human sP-selectin/CD62P (ng/mL)	23.3 ± 5.5	24.7 ± 4.8	0.57
D-dimer (mg/L)	0.07 ± 0.07	0.07 ± 0.06	1.00
vWF (IU/mL)	1.24 ± 0.35	1.27 ± 0.40	0.69

**Figure 4:**
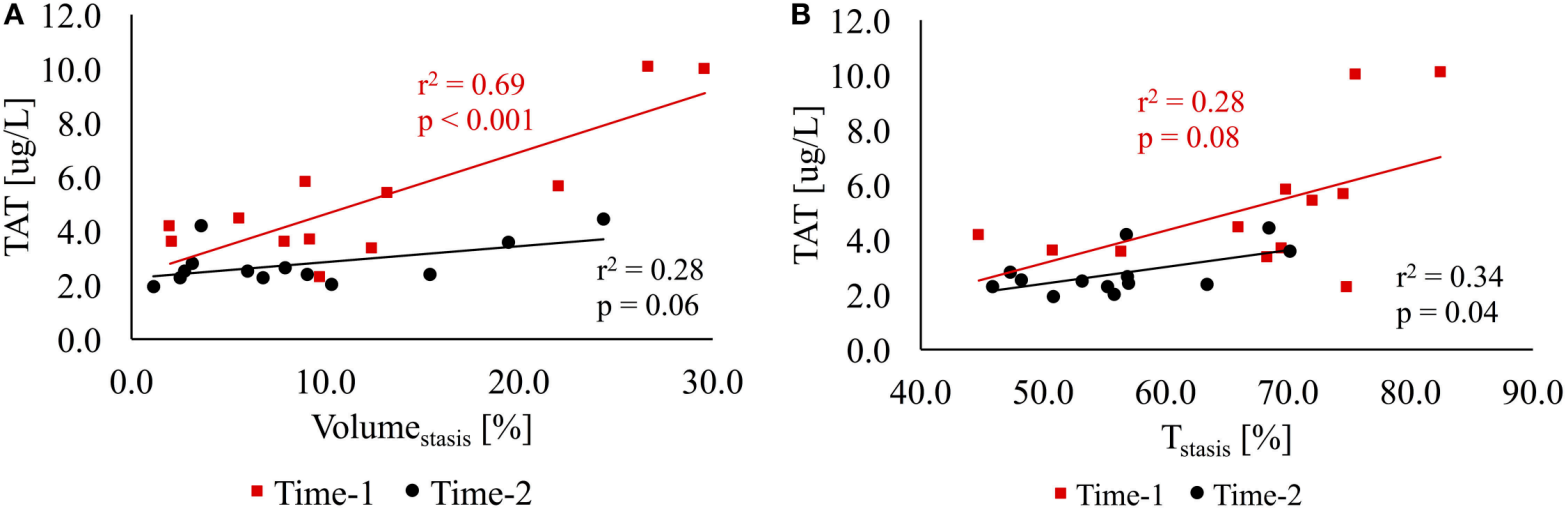
**(A)** The linear regression analysis between TAT and volume of stasis (Volume_stasis_) as a percentage of the LA volume at Time-1 (red) and Time-2 (black). **(B)** The linear regression analysis between TAT and duration of stasis (T_stasis_) as a percentage of the cardiac cycle at Time-1 (red) and Time-2 (black).

We found a significant linear correlation between TAT and Volume_stasis_ (*r*^2^ = 0.69, *p* < 0.001) at Time-1. At Time-2, the correlation between TAT and Volume_stasis_ was within borderline significance (*r*^2^ = 0.28, *p* = 0.06). Figure 4A shows the correlations between TAT and Volume_stasis_ at Time-1 and Time-2. We found a linear correlation with borderline significance between TAT and T_stasis_ (*r*^2^ = 0.28, *p* = 0.08) at Time-1 and a significant linear correlation between TAT and T_stasis_ (*r*^2^ = 0.34, *p* = 0.04) at Time-2. Figure 4B shows the correlations between TAT and T_stasis_ at Time-1 and Time-2.

## Discussion

In this study, the aim was to investigate the evolution of LA 4D blood flow patterns and hemostasis in patients following electrical cardioversion of AF. The strength of the study lies in the intra-group comparison of 4D flow CMR measurements over time and the assessment of their relation to the hemostatic markers. The findings showed abnormal 4D flow patterns in LA stunning that recovered after restoration of LA mechanical function. Further, there was a significant relationship between parameters of LA blood stasis and markers of hypercoagulability during LA stunning.

Atrial stunning after electrical cardioversion of AF causes transiently impaired atrial and ventricular function. The maximum change in LA volume within one cardiac cycle increases after restoration of LA mechanical function which could be an index of reverse remodeling of the LA. Our findings based on comprehensive assessment of time resolved multidimensional blood flow using 4D Flow CMR, showed increased LA blood stasis during LA stunning, compared to the restored LA mechanical function, indicating that a higher proportion of the LA blood volume had very low velocities, and that these low blood velocities occurred for a longer time period throughout the cardiac cycle. A decrease in LA blood stasis with recovery of LA mechanical activity is expected, however, to our knowledge, it has not been assessed using multidimensional flow-specific methodology before. The LA blood stasis parameters were calculated from 4D flow MRI data, by registering images temporally to create a motionless heart, a technique used recently (Lantz et al., 2016). In addition, we observed that the absence of atrial mechanical contraction hindered the rotating pattern of blood flow in the LA, resulting in low mean vorticity and vorticity near atrial wall during LD.

Recent studies have used 4D Flow CMR to evaluate LA blood flow in patients with AF or a history of AF. Fluckiger et al. quantified LA blood flow in patients with a history of AF using 4D Flow CMR and found no significant difference in blood flow between patients with history of AF and age-matched controls (Fluckiger et al., 2013). Lee et al. investigated the associations between LA flow and the clinical risk score CHA_2_DS_2_-VASc which is used to assess thromboembolic risk in AF patients (Lee et al., 2016). In this study, they found a significant inverse relationship between CHA_2_DS_2_-VASc score and mean and peak LA velocity in patients with a history of AF. Markl et al. studied patients with both AF and a history of AF, and found an association between impaired LA flow, defined as reduced velocity and increased blood stasis, LA volume and age (Markl et al., 2016c). The LA flow mean and peak velocities we report are within the range of the values reported in recent studies in patients with both AF and a history of AF (Markl et al., 2016a,c). In our study, we set the velocity threshold for stasis parameters as 15 cm/s which was within the range of velocity thresholds (10–18 cm/s) that can differentiate LA stasis (Markl et al., 2016c). In the study by Markl et al. the stasis duration as a fraction of the cardiac cycle was ~70% for the threshold velocity of 15 cm/s in patients with a history of AF, which is consistent with our findings (Markl et al., 2016c).

Hemostatic markers in patients post-electrical cardioversion of atrial fibrillation have been investigated in the previous studies, and the majority of findings are in agreement with the current findings. Freestone et al. (2006) studied patients that were fully anticoagulated with Warfarin, and found a tendency of lower (130 ± 48 vs. 157 ± 50 IU/dL) vWF at 4 weeks compared to at 2 h post-cardioversion. However, Li-Saw-Hee et al. performed a similar study and did not find any difference in vWF or soluble P-selectin between pre-cardioversion and post-cardioversion 3 weeks and 3 months (Li-Saw-Hee et al., 2001). Sakurai et al. (2004), studied patients up to 7 days post-electrical cardioversion of atrial flutter. Two-thirds of the patients had Warfarin (INR 1.5±0.7). The authors demonstrated increased levels (compared to normal values) of TAT (5.2 ± 4.5 μg/L) at 3 days post-cardioversion that are similar to the TAT levels we observe at 2–3 h post-cardioversion. Jacob et al. (2004), studied AF patients that were fully anticoagulated with Warfarin, and did not find any change in D-dimer between 2 h and 1 month post-cardioversion. In an interesting study, Lip et al. (1995), compared D-dimer levels between AF patients that were fully anticoagulated with Warfarin or had only received intravenous heparin. They showed that D-Dimer levels did not change between 3 days and 14 days post-cardioversion in patients that were fully anticoagulated. However, in patients who did not receive anticoagulant therapy, D-dimer levels were higher at 3 days compared to at 14 days post-cardioversion. To the best of our knowledge, the current study was the first to assess hemostatic markers and their correlation to 4D LA blood flow in atrial fibrillation patients who received new oral anticoagulants (NOAC) therapy.

Since, thrombus formation in the LA of patients with AF has been associated with blood stasis and abnormal intra atrial blood flow (Watson et al., 2009; Iwasaki et al., 2011), we investigated the relation between advanced measures of LA flow patterns and hemostasis. An elevated level of TAT concentration was found at atrial stunning, compared to the restored LA mechanical activity, which was significantly associated with volume and duration of LA blood stasis at atrial stunning. The significant association between volume and duration of stasis and TAT during atrial stunning suggests that impaired LA mechanical function leads to intra-atrial blood stasis that may play a role in LA thrombus formation. These findings suggest additional pathophysiological understanding of LA thrombus formation.

The findings of this study may lead to improved patient management following electrical cardioversion of atrial fibrillation. The comprehensive assessment of time-resolved volumetric LA blood flow using 4D Flow CMR enables better understanding of the link between multidimensional flow measures, such as stasis, and hypercoagulability, which might ultimately lead to optimization of duration and dosing of the anticoagulant therapy for each individual patient.

## Limitations

Beat to beat variations in LA flow were not assessed as 4D Flow CMR represents the average of velocities over many heart beats. Nevertheless, we only included patients with persistent AF that underwent electrical cardioversion, and hence, had sinus rhythm during the presence of atrial stunning. The VENC in the 4D Flow CMR acquisition was set to cover the velocity range in the left atrium and the left ventricle, hence, a slightly lower VENC could have been favorable for measurements of intra-atrial blood flow velocities. The number of patients included in this study was rather low. However, many of the key findings were based on intra-group comparison over time and the statistical analysis resulted in acceptable power. In this study, a separate analysis of LAA flow, which plays a role in the thromboembolic process, was not performed. Nevertheless, a recent study by Markl et al. showed that LAA peak velocities based on Doppler transoesophageal echocardiography are associated with 4D Flow CMR based metrics of LA velocities and LA stasis (Markl et al., 2016b). Hence, it is reasonable to believe that the analysis of LA 4D flow also provides insights into flow patterns that are pivotal to the thromboembolic process. The analysis of LAA 4D flow is subject to further studies. The use of anticoagulant treatment may alter the studied hemostatic markers, but we do not expect any effects of the anticoagulant treatment on the blood flow patterns.

## Conclusion

In this longitudinal study, LA multidimensional blood flow was altered and blood stasis was elevated during LA stunning compared to the recovered LA mechanical activity. Blood coagulation markers were also elevated during atrial stunning compared to the restored atrial mechanical function. The association between blood stasis and hypercoagulability proposes that assessment of LA 4D flow can add to the pathophysiological understanding of LA thrombus formation in patients with AF related atrial stunning.

## Ethics statement

This study was carried out in accordance with the recommendations of Regional Ethical Review Board with written informed consent from all subjects. All subjects gave written informed consent in accordance with the Declaration of Helsinki. The protocol was approved by the Regional Ethical Review Board (Dnr 2014/414-31).

## Author contributions

TE, LK, and C-JC conceived and designed the study. LK recruited the patients; LK and C-JC were involved in data collection; MC analyzed the data, drafted the manuscript, and prepared figures; MC, TL, LK, and C-JC interpreted the results; C-JC edited the manuscript critically. TL, TE, and LK revised the manuscript. All authors read, approved the final manuscript, and agreed to be accountable for all aspects of the work in ensuring that questions related to the accuracy of integrity of any part of the work are appropriately investigated and resolved.

### Conflict of interest statement

The authors declare that the research was conducted in the absence of any commercial or financial relationships that could be construed as a potential conflict of interest. The reviewer ML and handling Editor declared their shared affiliation.
